# Metabolic classification of microbial genomes using functional probes

**DOI:** 10.1186/1471-2164-13-157

**Published:** 2012-04-27

**Authors:** Chi-Ching Lee, Wei-Cheng Lo, Szu-Ming Lai, Yi-Ping Phoebe Chen, Chuan Yi Tang, Ping-Chiang Lyu

**Affiliations:** 1 Institute of Bioinformatics and Structural Biology, National Tsing Hua University, Hsinchu, Taiwan; 2 Department of Computer Science, National Tsing Hua University, Hsinchu, Taiwan; 3 Department of Biological Science and Technology, National Chiao Tung University, Hsinchu, Taiwan; 4 Department of Computer Science and Computer Engineering, La Trobe University, Melbourne, Australia; 5 Department of Computer Science and Information Engineering, Providence University, Taichung, Taiwan; 6 Department of Medical Science, National Tsing Hua University, Hsinchu, Taiwan; 7 Graduate Institute of Molecular Systems Biomedicine, China Medical University, Taichung, Taiwan

## Abstract

**Background:**

Microorganisms able to grow under artificial culture conditions comprise only a small proportion of the biosphere's total microbial community. Until recently, scientists have been unable to perform thorough analyses of difficult-to-culture microorganisms due to limitations in sequencing technology. As modern techniques have dramatically increased sequencing rates and rapidly expanded the number of sequenced genomes, in addition to traditional taxonomic classifications which focus on the evolutionary relationships of organisms, classifications of the genomes based on alternative points of view may help advance our understanding of the delicate relationships of organisms.

**Results:**

We have developed a proteome-based method for classifying microbial species. This classification method uses a set of probes comprising short, highly conserved amino acid sequences. For each genome, *in silico *translation is performed to obtained its proteome, based on which a probe-set frequency pattern is generated. Then, the probe-set frequency patterns are used to cluster the proteomes/genomes.

**Conclusions:**

Features of the proposed method include a high running speed in challenge of a large number of genomes, and high applicability for classifying organisms with incomplete genome sequences. Moreover, the probe-set clustering method is sensitive to the metabolic phenotypic similarities/differences among species and is thus supposed potential for the classification or differentiation of closely-related organisms.

## Background

Owing to new sequencing technologies, the number of microorganisms with completely or partially determined genomic sequences is rapidly increasing, inclusive of many species that cannot be artificially cultured and many new/unknown species collected from environmental samples. As the amount of genomic information increases, interdependent relationships between species (e.g., the symbiotic partnership between bacteria and host) and the survival strategy of certain microbes and their living environment (e.g., *Archaea *and *Bacteria *living in the hot spring) become particularly interesting. As a result, the microbiology field has gradually expanded its focus from microbial clones to microbial communities, and some new research fields have accordingly formed, such as metagenomics [1]. Because species are no longer studied through clonal isolates, the first question encountered by microbial genomics researchers looking at a heterogeneous population is often "Who is there?". A number of short biomarkers, such as the 16S rRNA genes, exhibit detectable sequence variations in a basically conserved framework between species and can be used both to identify individual species within a community and to infer their phylogenetic relationships [2-9]. However, there are drawbacks in analysis of short sequences. These markers generally comprise just a small proportion of an organism's genome; for example, 16S rDNA contributes less than 0.2% of the bacterial genome [10]. Previous studies have suggested that the lack of metabolic information in this small amount of genetic material renders it insufficient to describe the way of life for an entire organism or species [10,11].

Many studies have used existing metabolic databases, e.g., Kyoto Encyclopedia of Genes and Genomes (KEGG), to understand metabolic relationships between organisms and to construct complete relationship trees [12,13]. Interestingly, although relationship trees constructed using metabolic data are generally consistent with existing phylogenetic trees; there are important differences in the details [14-17]. In some ways, these relationship trees more effectively explain the survival strategies that organisms have developed to handle unique metabolic relationships, such as how symbiotic bacteria share metabolites with hosts [14,18,19]. However, these methods still have many basic shortcomings, including the dependence on complete quantitative information of metabolites, difficulty in defining reactants and intermediates, heavy reliance on human annotation, and the requirement to deal with excessively complex metabolic data [20]. Many of the problems associated with constructing organismal relationships from metabolic data may be avoided by using a proteomic approach. Since proteins are the basic functional units of biological systems, construction of proteomic trees may prove effective in describing the metabolic relationships between species and in reconstructing phylogenetic relationships [21-25].

Microorganisms were chosen as the initial test subjects for our proteome-based classification method. Distinct from complex multicellular organisms, most microorganisms are unicellular and structurally simple. In addition, most microbial intracellular proteins react directly to external stimuli. While there are differences among related proteins, functionally critical protein domains are typically very conserved [26]. This study used a common set of conserved protein sequences -- which is called a probe-set -- to determine relationships between organisms, an analysis that could be labelled "seeking commonality among variation". Then, "seeking variation among commonality", differences in conserved sequences between organisms were used to categorize individual organisms. Currently, the genomes of more than one thousand microorganism strains have been sequenced [27,28]. The habitats of these microorganisms vary greatly, from symbiotic environments to extreme ecosystems. Since ample biochemical and metabolic data concerning these microorganisms are available, they were selected for the initial development and evaluation of our method.

We first verified that the proposed probe-set method could identify differences between enzymes and between metabolic pathways. Next, we demonstrated that the method could accurately differentiate host-associated from free-living bacteria. Finally, using sequence data from hundreds of microorganisms, we constructed a large-scale relationship tree. Several factors contribute to the success of the probe-set method for clustering microorganisms that share unique metabolic relationships, that coexist in extreme environments, or that possess extraordinary metabolic capabilities (e.g., green sulfur and photosynthetic bacteria). The probe-set method is essentially a compositional analytical method that avoids the disadvantages of conventional sequence-based classification methods, including the difficulty of classifying sequences containing exchanges or recombinations [29-32]. An important advantage of the probe-set method is its ability to classify organisms whose genomes have not been sequenced. This should make this method feasible for metagenomics studies, which generally involve incomplete and poorly annotated genomic sequences [33]. In addition, the proposed method is able to detect metabolic differences between organisms with very close evolutionary relationships. Finally, to compare trees generated by the probe-set method with other kinds of classification trees, a tree topology comparison method is developed in this work. This tree comparison method is useful for large scale phylogenetic tree comparing and can be a standard evaluating approach for further tree building methods.

## Results

### Probe-set method: definition and evaluation

#### The probe-set concept

We used short, highly conserved, amino acid sequence fragments to define the proteomic probe-set. For implementation, the Prosite descriptors (peptide fragments mainly ranging from 10 to 20 residues in length) were selected as probes in this work. Each Prosite descriptor represents a conserved protein sequence extracted from a number of proteins with similar biological functions. A descriptor can be considered a consensus of a group of text strings that occur frequently at the functional site(s) of a certain kind of enzymes, transporters, receptors, etc. For example, descriptor PS00100, which represents the sequence pattern commonly found at the active site of chloramphenicol acetyltransferase, can be written as Q-[LIV]-H-H-[SA]-x(2)-D-G-[FY]-H. Sequences such as Q-L-H-H-S-G-G-D-G-F-H and Q-V-H-H-A-G-G-D-G-Y-H match this pattern descriptor, or probe. If a probe is found in a protein, that protein generally has the function represented by the probe. Although a single probe only describes a small fraction of the functions of a proteome/genome, a reasonable representation can be obtained by using a sufficiently large collection of probes. This is the core concept of the proposed method, which utilizes approximately 1,000 probes. To determine relationships among a chosen set of organisms, probe frequency patterns of the 1,000 probes were calculated for each proteome. These frequency patterns were then clustered to construct the classification tree. The probe-set method is thus a global-scale method that disregards noise in the system (i.e., non-conserved protein sequences) and focuses on conserved protein sequences that more accurately describe an organism's functional capacities.

#### Protein-level evaluation

**Figure 1:**
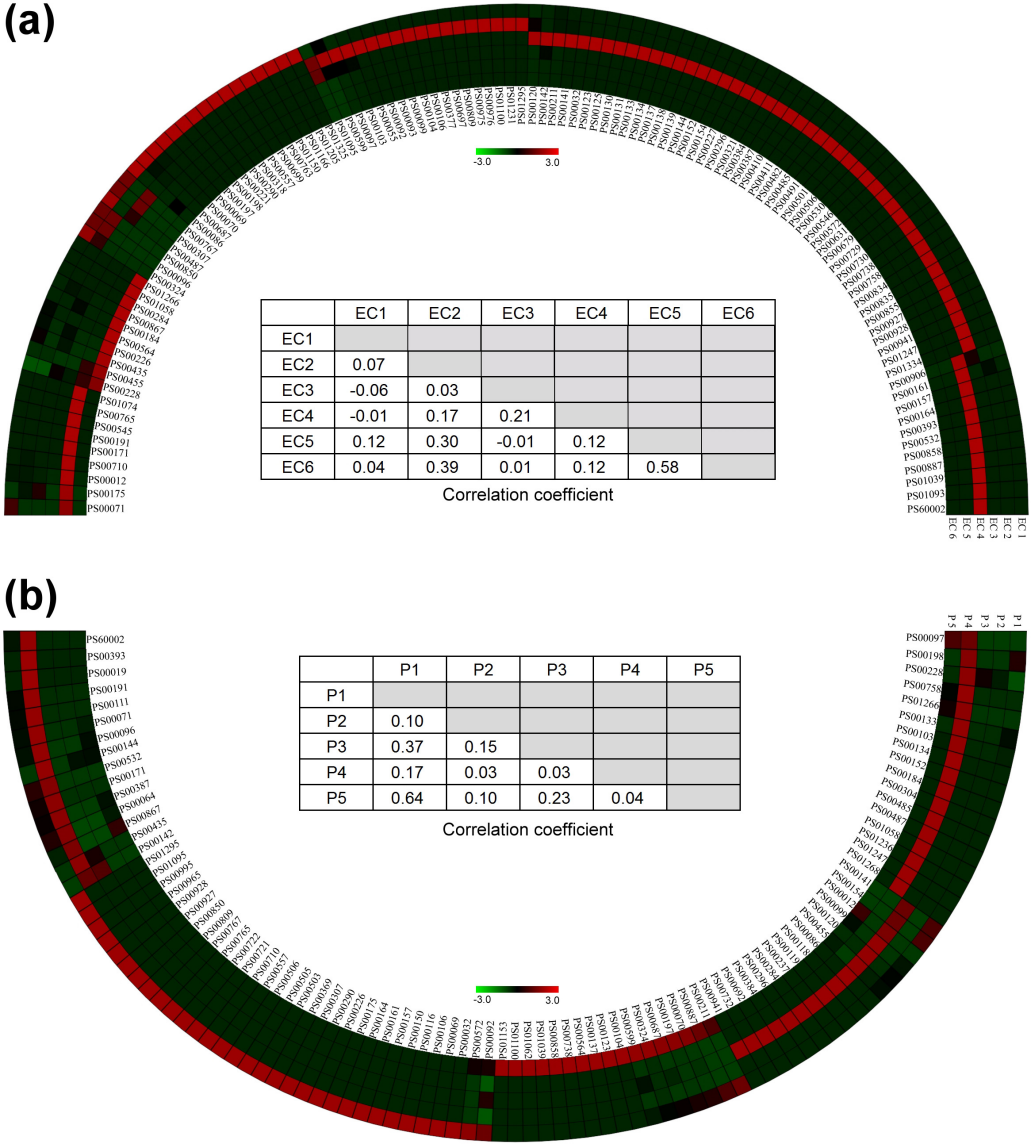
**Frequency patterns of six enzyme categories and five metabolic pathways**. Probe-set frequency pattern and correlation coefficient of: (a) EC Level 1 protein collectives (EC1: oxidoreductases, EC2: transferases, EC3: hydrolases, EC4: lyases, EC5: isomerases, and EC6: ligases); and (b) five common metabolic pathways (P1: carbohydrate metabolism, P2: energy metabolism, P3: lipid metabolism, P4: nucleotide metabolism, and P5: amino acid metabolism). The probe-set frequencies are normalized and represented by the gradient color in the frequency patterns, shown by the half circles. The inner tracks of the circles represent probe names, and the color cells of the outer tracks represent the normalized probe-set frequency values. The table in each panel respectively records the correlation coefficients between the protein collectives. In this figure, probes with zero frequency values are omitted.

As a first test of the probe-set method, we focused on enzymes with well-defined functions. The Enzyme Commission (EC) has hierarchically categorized enzymes using a system consisting of four levels. The classification of each enzyme is represented by a four-digit number representing the four levels (for example, 1.2.3.4 denotes oxalate oxidases). The first EC level is a rough functional classification consisting of six classes: oxidoreductases (EC1), transferases (EC2), hydrolases (EC3), lyases (EC4), isomerases (EC5), and ligases (EC6). Enzymes within the same level 1 class perform similar chemical reactions, but can have large differences in sequence and structure. In addition, their target substrates and reactants may be very different. Based on these "existing variations among commonality", we chose the first EC level of enzyme classes as the first data set for evaluating the probe-set method. A non-redundant set of 2,935 enzymes was created by randomly selecting one enzyme from each level 4 EC classification. Probe-set frequency patterns for the six EC level 1 classes are shown in Figure 1a. To visually represent the results, frequency patterns were color-coded and scaled by brightness (see Methods). Correlation coefficients (CC) were calculated to compare probe-set frequency patterns. Very clearly, each EC class had a unique probe frequency pattern, and the CC values between classes were generally low. Although enzymes within a single EC class might have differences in sequence and structure, differences between classes were even larger, as they include diverse catalytic mechanisms, substrates, and cofactors. These results demonstrate that the probe-set method can distinguish between groups of proteins with fundamental functional differences, and that it may be computationally feasible to develop an automated proteome classification procedure.

#### Pathway-level evaluation

It is perhaps not surprising that enzymes with different functions have different probe-set frequency patterns. We next asked whether entire metabolic pathways could be distinguished using this method. This task is inherently more challenging and more relevant, as pathways are not composed of proteins with the same function, but of proteins with multiple functions that act cooperatively. For example, according to the KEGG database (the most extensive pathway database at present), a typical glycolysis/gluconeogenesis pathway requires 49 enzymes, including 15 EC1, 14 EC2, 6 EC3, 4 EC4, 8 EC5, and 2 EC6 enzymes. Five major metabolic pathways are commonly shared among organisms: the carbohydrate (P1), energy (P2), lipid (P3), nucleotide (P4), and amino acid (P5) pathways. As shown in Figure 1b, different metabolic pathways possessed different probe-set frequency patterns, and the CC values between pathways were low. These data indicate that the probe-set method can effectively discriminate between major metabolic pathways. It is noticeable that metabolic reactants and intermediates were not considered in this analysis; therefore, the discriminatory power of the probe-set method would not be significantly affected by unclear definitions of reactants and intermediates (see Background).

#### Proteome-level evaluation

**Figure 2:**
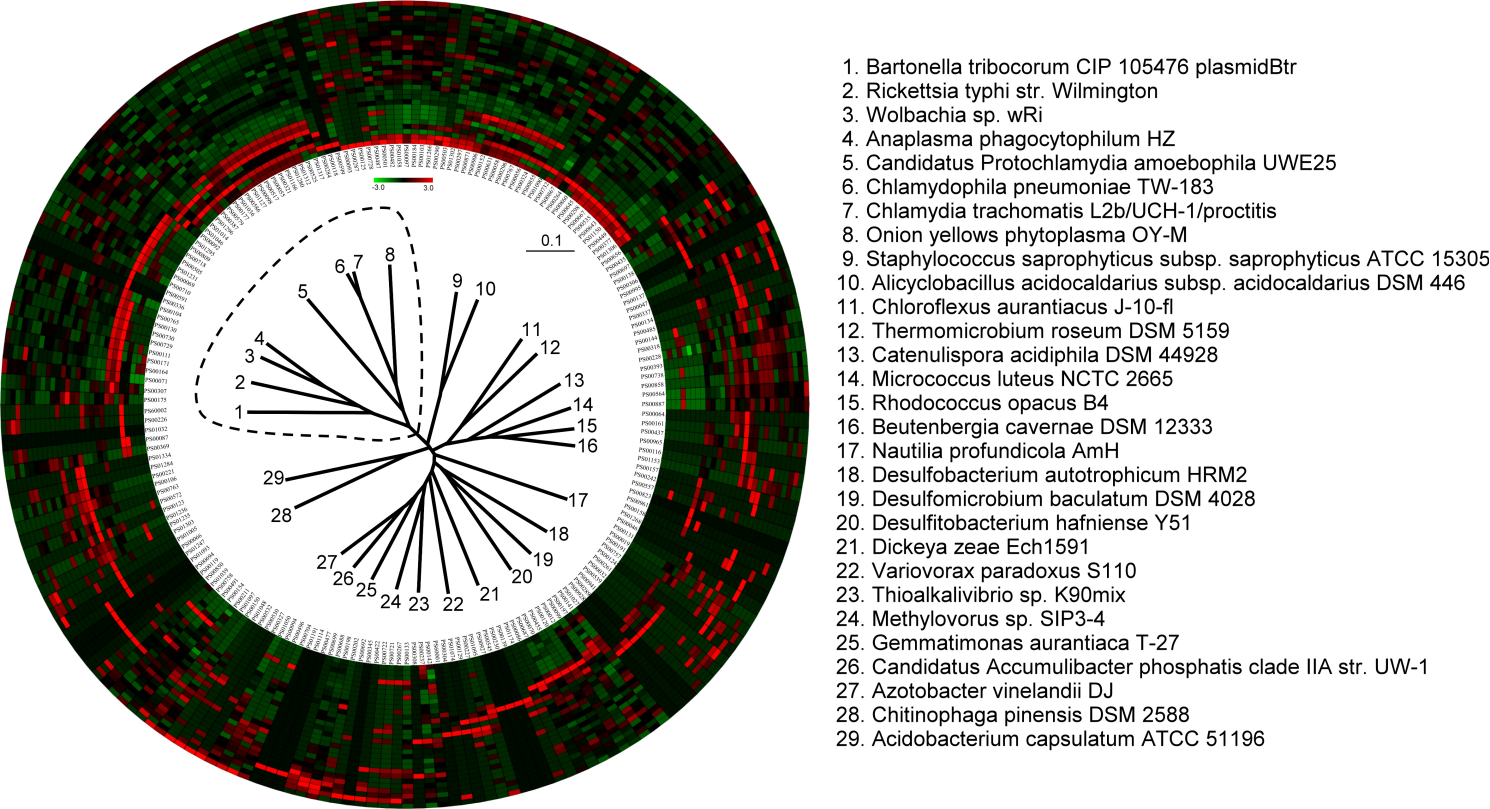
**Probe frequency patterns and clustering result of 29 free/host-living microorganisms**. The frequency patterns are represented by the circle; the inner track indicates the probes that can be found in the proteome of these species. These microorganisms, including 8 host-living and 21 free-living species, were clustered according to the similarities of their probe-set frequency patterns by using the CLUSTER 3.0 program, which also generated the classification tree positioned in the center. The host-living and free-living microorganisms are clearly separated. The cluster encircled by the dotted line consists of the 8 host-living species, which correspond to the 8 innermost colored tracks. The positioning of the colored tracks is also determined by the clustering program CLUSTER 3.0.

So far we have shown that different categories of enzymes and metabolic pathways possess well-distinguishable probe-set frequency patterns. However, relying solely on enzymes to categorize proteomes is inadequate, as organisms require many non-enzymatic proteins to survive, such as those involved in signal transduction or transcriptional regulation. In fact, the five metabolic pathways used in the previous analysis contain polypeptide sequences recognized by only around 100 probes (10% of the probe set). To more comprehensively test the method's capability, entire proteomes of both host-associated and free-living microorganisms (as annotated in the GOLD database) were examined by constructing a probe-set based classification tree (see Methods for the way we constructed classification trees). Figure 2 clearly shows that host-associated and free-living organisms were differentiated using the probe-set method. Both the color-coded probe frequency patterns and the classification tree revealed considerable differences between the two groups of organisms. The disparity in living environments between the two groups has perhaps leaded to the utilization of different metabolic pathways, allowing probe-set clustering to differentiate successfully between the two groups.

**Figure 3:**
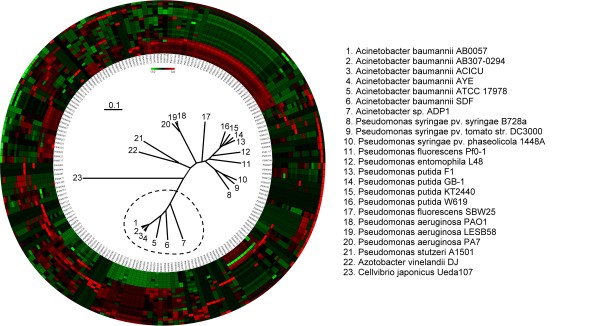
**Proteomic tree of 23 class *Pseudomonadales *species**. The probe-set frequency patterns of 23 species belonging to class *Pseudomonadales *are shown by the clustered color tracks. Most species of this class are free-living microorganisms; however, genus *Acinetobacter *is an exception. *Acinetobacter *species are mainly host-associated. The classification tree and the clustered color tracks show that the *Acinetobacter *species, which are encircled by the dotted line and correspond to the 7 innermost color tracks, form a distinct cluster from other *Pseudomonadales *species.

Previous studies have found that host-associated microbes often lack certain metabolic pathways. The reason for this involves shared metabolites, such as the ability to obtain non-essential amino acids from the host [34,35]. Most organisms of the class *Pseudomonadales *are free-living microbes; however, organisms of the genus *Acinetobacter*, which also belongs to class *Pseudomonadales*, are mainly host-associated microbes. As shown in Figure 3, the *Acinetobacter *organisms were all clustered in the same subgroup that is distant from other organisms of *Pseudomonadales *in the probe-set classification tree. Over time, host-associated organisms adapt to the living environment and alter/lose metabolic capabilities to better co-exist with their host organisms [36]. These changes would be expected to significantly alter the probe-set frequency pattern and therefore allow the proteome-based method to differentiate host-associated from free-living microorganisms.

### Global proteomic tree

**Figure 4:**
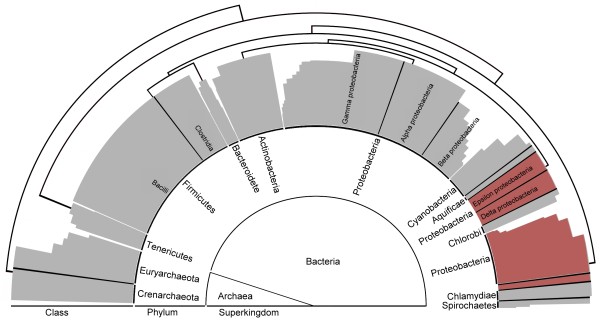
**Large-scale proteomic tree**. This is a simplified version of Additional file 1, a large-scale proteomic tree of 843 microbes. Only major phyla and classes are shown here. Most species are clustered in accordance with their taxonomic classifications. The red sectors highlighted the species situated differently from their taxonomic positions. *Epsilon proteobacteria *and *Delta proteobacteria *are mainly chemosynthesis organisms. Although they belong to the *Proteobacteria *phylum, they are clustered with *Cyabobacteria, Aquificae *and *Chlorobi*, which are species with unique ways of energy production. The other *Proteobacteria *species are mainly parasitic organisms; they are clustered with *Chlamydiae *and *Spirochaete*, which are parasitic species as well. The clustering results of these species demonstrate that the probe-set method tends to cluster organisms according to their metabolic or phenotypic similarities, especially at lower taxonomic levels.

We next sought to thoroughly evaluate the proposed classification method and compare our proteomic tree with the standard phylogenetic tree. A large-scale clustering of 843 microbial species was performed using the probe-set method. As shown in Additional file 1 (see also Figure 4 for a simplified tree to the class level), the majority of probe-set classification results were consistent with existing taxonomic classifications. For example, *Archaea *and *Bacteria *were separated. Besides, species were mostly grouped in accordance with their phylum and class taxonomic ranks. As for lower taxonomic levels, such as order and family, the classification results of our method also correlate well with regular taxonomic ranks (Additional file 1). However, there are still several groups exhibiting significant differences from traditional phylogenetic classifications.

Within the bacteria superkingdom, the *Proteobacteria *phylum was divided into two groups by the probe-set method (see the red sectors in Figure 4). This grouping is different from regular taxonomic classifications, according to which *Proteobacteria *species should be clustered in the same group. The first group consisted of organisms mainly from phyla *Proteobacteria, Spirochaete*, and *Chlamyidae*; most organisms in this group have parasitic/symbiotic characteristics. The second group comprised many species characterized by their unique ways of energy production, such as photosynthesis (*Cyanobacteria *and *Green sulfur bacteria*) and chemosynthesis (chemoautotrophs, chemolithoautotrophs, and hydrogen sulfur oxidization species). These results imply that, at taxonomic ranks lower than class, the probe-set method tends to classify species according to their biological and metabolic characteristics. Since, in the example of *Proteobacteria*, the characteristics were acquired due to the living environments of the organisms (e.g., hot-spring water), it is expected that our classification method can help identify organisms living in similar environments and provide information about how they survive in and interact with their environments.

As expected, in the *Archaea *superkingdom (see the lower part of Additional file 1), we also found that the similarities of living environment exerted important effects on the classification results of our method. Phylum *Euryarchaeota*, for instance, were divided into two major groups that were consistent with the taxonomical classes of those organisms (e.g., the *Crenarchaeota *class and *Euryarchaeota *class), but the grouping of organisms in each class did not exactly follow traditional taxonomic classification; instead, halophiles (living in high salt concentration environment), thermophiles (living in high temperature environment) and methanophiles (using methane as carbon and energy source) were respectively clustered together.

From these results, the probe-set method is able to reconstruct traditional phylogenetic classifications from a proteomic perspective and detect non-phylogenetic commonalities in organisms that have adapted unique biochemical capabilities. We believe that this is because the conserved sequences of a proteome can reflect the biological characteristics of an organism more accurately than genomic DNA.

### Metabolic classifier for closely related species

**Figure 5:**
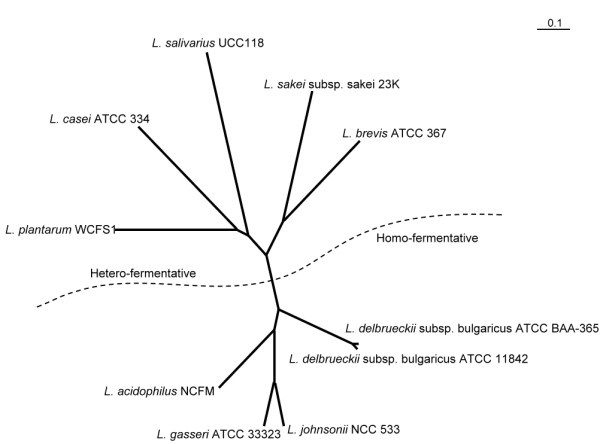
**Proteomic tree of ten genus *Lactobacillus *species**. There are ten *Lactobacillus *species known to have fermentative abilities according to Bergey's Manual of Systematic Bacteriology [6]. Some of them obtain energy through homo fermentation while others through hetero fermentation. Since these species are very closely-related, traditionally, biochemical analyses would be performed to distinguish or classify them. By using the proposed probe-set method, these species are clustered into two groups. As indicated by the dotted line, these two groups respectively consist of homo and hetero fermentative species. This result demonstrates the feasibility of the probe-set method as a metabolic classifier, which is especially useful for classifying closely-related species that are difficult to distinguish by biomarkers.

Traditional phylogenetic analyses based on short biomarkers may not be sensitive enough to differentiate organisms with highly close evolutionary relationships [37]. In traditional taxonomy, metabolic characteristics of organisms derived from biochemical and metabolic analyses are often used to help differentiate closely-related species [38]. However, analyses such as these are time-consuming and expensive. If bioinformatics techniques could be used in this process, the identification and classification of microorganisms would be much easier. We supposed that the probe-set method, which effectively classifies organisms according to their metabolic differences, is applicable in this regard. For instance, there are two types of lactic acid fermentation activities, i.e., the homo-fermentation and hetero-fermentation, evolved in the genus *Lactobacillus*, that is, lactic acid bacteria fermenting lactose into lactic acid. As of the date of this article, there have been 10 species in the genus Lactobacillus possessing known fermentative capability as annotated in the GOLD database [27,39]. Classification results of these 10 species by the proposed probe-set method clearly revealed that they belong to two different groups: the hetero- and homo-fermentive groups (Figure 5). Very probably, the proteomes associated with these two kinds of fermentative activities are different, allowing the probe-set method to distinguish between them. Since the metabolic variances of these *Lactobacteria *cannot be determined by 16S rRNA homolog analysis but only through metabolic analysis [40], this example demonstrates that the proposed method may be applied to finely classify organisms with such close evolutionary relationships that traditional phylogenetic analyses are inadequate to differentiate.

### Phylogenetic classifier for metabolic related species

**Figure 6:**
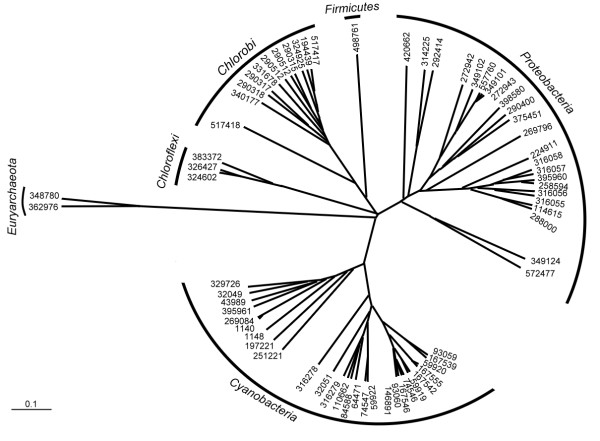
**Proteomic tree of species with similar metabolic capacities**. There are 67 photosynthetic microbes recorded in the GOLD database [27]. These microbes belong to six different phyla. In this probe-set classification tree, each organism is represented by its taxonomic ID. These microbes are clustered into six groups, each of which precisely corresponds to a phylum, as indicated by the labels at the outer tracks. This example illustrates that the probe-set method can well reveal the phylogenetic relationships among species with highly similar metabolic capabilities.

To see whether our method could recognize the phylogenetic relationships among organisms possessing similar metabolic or phenotypic characteristics, we constructed a classification tree for various microbes capable of photosynthesis (according to the annotation by the GOLD database). Though all harvesting energy from light, these 67 organisms belong to six different phyla. As shown in Figure 6, clearly, microbes in the same phyla are clustered together. Since there were no non-photosynthesizing microorganisms serving as references for our classification method, the results demonstrated that metabolic or phenotypic similarities would not blurred the focus of our probe-based classifier -- which would first classify microorganisms according to their phylogenetic relationships at higher classification levels and then distinguish them based on their functional similarities at finer levels.

### Influences of horizontal gene transfer on classification quality of the proposed method

**Figure 7:**
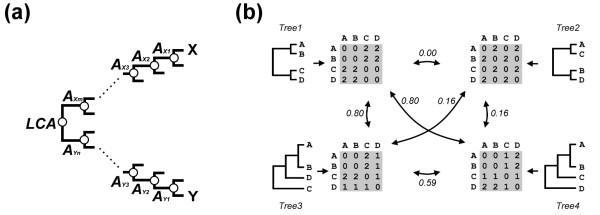
**Measurement of the similarity between classification trees**. (a) Computation of the traveling distance between leaves. In a classification tree, between any two species there exists a lowest common ancestor (LCA) and, a species can be considered as a leaf. Given a pair of leaves *X *and *Y*, if a worm starts from *X *and travels along the shortest path between the two leaves while another worm starts from *Y *and travels along the same path, before they meet at the LCA node, the first worm needs to go through *m *ancestor nodes of *X *(*A_Xm_*) and the other worm needs to go through *n *ancestor nodes of *Y *(*A_Ym_*). In this example, the traveling distance between *X *and *Y *is calculated as *m *+ *n*. (b) Similarity between some sample trees. A tree can be transformed into a distance matrix after computing all the leaf-to-leaf traveling distances. This figure exhibits the distance matrices of four sample trees. The topological similarity between two trees is then computed as the correlation coefficient between the distance matrices of the trees; a high correlation coefficient indicates a high similarity.

Due to the dynamic nature of microbial genomes, horizontal gene transfer (HGT) often occurs [25,41-43]. As a result, proteome-based strategies to build classification trees may be vulnerable to the influence of foreign genes. To determine to what extent the HGT phenomenon would influence the classification results of our method, we compared two proteomic trees that it generated -- one contained HGT genes while the other did not. HGT DataBase is a database of microorganisms that bear HGT genes [44]. We first constructed a HGT-genes-containing probe-set classification tree, Tree HGT^+^, for all the 415 species recorded in the HGT DataBase (see Additional file 2a). Next, from the genomes of the same 415 species we removed all potential HGT genes determined by the HGT DataBase. These HGT-genes-filtrated genomes were subjected to our tree-construction procedure and produced Tree HGT^- ^(Additional file 2b). Since there is no convenient method for comparing the similarities between large classification trees like Tree HGT^+ ^and Tree HGT^-^, we developed a method to quantitatively describe the topological similarity between two classification trees as a correlation coefficient (CC) of their leaf-to-leaf traveling distance matrices (Figure 7; see Methods for details). A high degree of similarity between trees results in a high CC value. The CC value for two exactly the same trees is 1. Theoretically, two randomly-constructed trees have a CC value of 0. The CC between Tree HGT^+ ^and Tree HGT^- ^was very high (0.93). As shown in Additional file 2, in which different colors were labelled to different groups of organisms, the grouping of organisms (i.e., the coloring patterns) on the HGT^+ ^and HGT^- ^trees are highly similar, implying that the existence or inexistence of HGT genes did not significantly influence the classification quality of the proposed method.

### Simulated testing of organisms with incomplete genomes

Genome sequencing methods often split contiguous sequences into thousands of fragments that must be recombined in the correct order according to overlapping regions. Reconstructing an entire DNA genome is not trivial and there are many publicly available genomic sequences still incomplete. Compositional analysis, which is the main concept behind the proposed probe-set method, represents a possible solution for analyzing incomplete genomic sequences. In this study, we simulated two scenarios to assess the reliability of the probe-set method in dealing with incomplete genomes.

**Figure 8:**
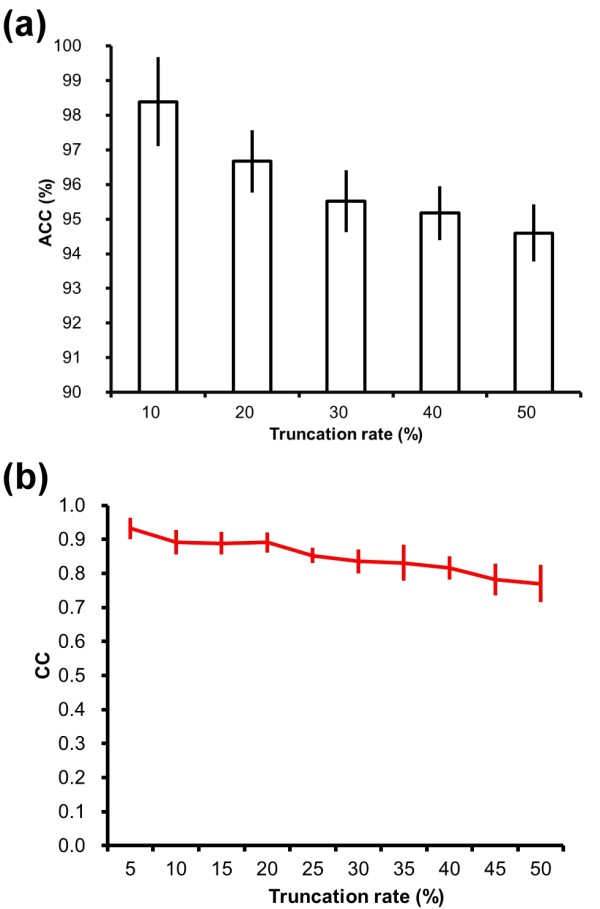
**Performance of the probe-set method in classifying incomplete genomes**. Using the taxonomy database provided by NCBI, three organisms were randomly selected from each class to perform this experiment. A native probe-set classification tree of the 87 selected organisms was first constructed. Then, the genomes of these organisms were randomly truncated in different extents. With the truncated genomes, new classification trees were constructed and compared to the native tree. (a) Accuracy for the reclassification of a randomly truncated genome. The 87 genomes were subjected to random truncation and tree reconstruction one genome at a time. The accuracy (the y-axis) is computed as the ratio of truncated genomes that did not affect the topology of the tree. At each truncation rate, as indicated by the x-axis, the experiment was repeated 10 times and the standard deviation was thus obtained. (b) Similarity between the native classification tree and the tree with large-scale random truncations. All the 87 genomes were subjected to random truncation at the same time in this experiment. After a random truncation, the tree was reconstructed and its correlation coefficient (y-axis) with the native tree is computed to measure its topological similarity to the native tree. At each truncation rate, as indicated by the x-axis, the experiment was repeated 10 times and the standard deviation was thus obtained.

First, we randomly selected three strains from each phylogenetic class of microorganism and constructed a reference classification tree of 87 species. For each species, we performed random truncation of its proteome (removing some proteins at random) before reconstructing a new classification tree. If the topology of the new tree was exactly the same as the reference tree, a precise classification was recorded. After every species was tested, the accuracy of reclassification was computed as the number of precise classifications divided by 87. At any given extent of truncation, this random truncation experiment was repeated 10 times to obtain the average and standard deviation values of the accuracy. The average accuracy values with various extents of truncation are illustrated in Figure 8a. Notably, even if only 50% of the genome was retained, an average accuracy of 94% could still be achieved, proving that the probe-set method has good fault tolerance.

Next, we performed a more stringent assessment, in which every of the 87 species was randomly truncated before reconstructing the classification tree. The topological similarity between the reconstructed tree and the reference tree was quantified as a CC value as described in the above subsection. As shown in Figure 8b, the probe-set method does not require full amount of information to obtain good classification results; the CC value for the reconstructed tree with 50% truncated genomes was ~0.78. The high accuracy and CC values obtained in these truncation tests imply that the probe-set method represents a potential and convenient tool for microbial taxonomy, particularly for species whose genomes have not been completely sequenced.

### An example of incomplete genome: *Leptospirillum ferrodiazotrophum*

**Figure 9:**
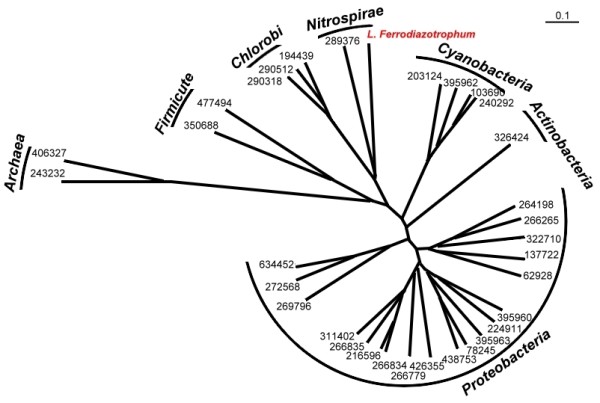
**Proteomic tree of 33 bacteria with nitrogen fixation capabilities**. The probe-set classification tree of the 33 nitrogen-fixing microorganisms recorded in the GOLD database [27]. Each leaf is labelled with the taxonomic ID of the organism, and the labels on the outer tracks indicate the phyla of the species. Similar to the result shown in Figure 6 for the photosynthesis microbes, the clustering of these nitrogen-fixing microorganisms is consistent with the taxonomic classification of these species. Notably, *L. ferrodiazotrophum*, the genome of which is only partially sequenced, is correctly clustered with *T. yellowstonii *(taxonomic ID: 289376), a species that belongs to the same phylum with *L. ferrodiazotrophum*, i.e., Nirtospirae.

*L. ferrodiazotrophum *is a small nitrogen-fixing bacterium living in acidic environments and using ferrous iron as the only electron donor to perform chemolithotrophic activities [45,46]. It was isolated from an environmental sample by a metagenomics project [46], and a fully sequenced genome of this strain is not available yet. Only 182 genomic scaffolds (i.e., segments of the genome sequence) and one 16S ribosomal RNA gene of this species could be downloaded from Genebank. Taken together, these scaffolds were ~2,804 Kb in length and contained 2,658 annotated protein sequences. The phylogenetic position of this species is phylum *Nitrospirae*, which contains only one completed genome sequence (*Thermodesulfovibrio yellowstonii *DSM 11347, a nitrogen-fixing, thermophilic and sulfate-reducing bacterium [47]). Figure 9 demonstrates the clustering results for all nitrogen-fixing bacteria recorded in the GOLD database. As expected, *L. ferrodiazotrophum*, even with its genome sequence incomplete, was clustered with *T. yellowstonii*, properly reflecting their closeness in phylogenetic relationship. Based on this real case, the probe-set method proved again its effectiveness in classifying species with incomplete genome sequences.

## Discussion

### Advantages of the probe-set method

#### A classification method with a combinative nature

Due to the immense diversity of microbial morphologies distributed in various living environments, classification strategies for the microorganisms based on wet-lab techniques may be costly, time-consuming and thus inefficient when compared with strategies based on computation. Classification methods with different properties and emphases have been proposed. Phenetic classification methods classify microbes according to measurable features such as cell shape, staining properties, and metabolic characteristics [38]. Proteomic comparisons based on two-dimensional polyacrylamide gel electrophoresis were also applied to distinguish closely-related species [48]. Biomarkers, e.g., the 16S rRNA genes, cytochrome c, and ATPases, are used as molecular clocks to elucidate the evolutionary history of species [38]. Approaches such as whole genome alignment [49] and gene ordering analysis [50] construct phylogenetic trees from a genomic point of view. The proposed probe-set method focuses on expressible information contained in the genome and thus its classification emphasizes the functional relationships among species. In order to comprehensively understand the evolutionary relationships among organisms, several attempts have been made to combine all existing classification methods, such as the polyphasic taxonomy introduced by Colwell [51] and refined by Vandamme et al. [38]. Polyphasic taxonomy researches classify organisms based on their phenotypes, genotypes, and chemotaxonomic characteristics and the results suggested that any single feature or biomarker is insufficient to properly classify organisms at every level of taxonomy [37]. Since the proposed probe-set method utilizes almost all currently known consensus protein sequence patterns to perform classification, its combinative nature may make it a good alternative and comprehensive way to classify microorganisms.

#### A classification revealing metabolic phenotypic similarities

In our large-scale proteomic tree, the positions of several species differed significantly from their positions in the traditional taxonomic classification tree, which is established according to the similarities of 16S rRNA genes. Compared with the classifications based on 16S rRNA genes, our method tends to classify organisms according to the similarities of their metabolic capabilities (see Results, Figure 2 and 3). A metabolic capability of an organism is made possible by the cooperation of many genes, the existence of which, as we supposed, may be revealed by the composition of genome of the organism. Indeed, as shown in Figure 1b, the probe-set compositions of various metabolic pathways can be very different. Since the proposed probe-set method classifies organisms according to their genomic compositional differences, this method should be able to detect the genomic differences correlated with the metabolic capabilities of organisms.

### Possible applications

#### Large-scale and whole genome-based clustering

The probe-set method compares genomes by transforming the coding sequences into probe frequency patterns and then clustering them. The clustering of these frequency patterns is much simpler than performing multiple sequence alignment of whole microbial genomes. The probe-set classification takes only 1.3 minutes to build a large proteomic tree containing 843 organisms on a computer with an Intel Xeon 2.13 GHz processor and 3 GB memory. This high speed makes the proposed method well applicable to perform large-scale classification of microorganisms. Moreover, the proposed method compares organisms based on information extracted from their whole genomes and thus considers more function-related characteristics than traditional phylogenetic analyzing strategies do. The example shown in Figure 5 has well demonstrated that *Lactobacteria*, the functional differences of which can not be detected by the traditional 16 rRNA-based method, are classified according to their fermentative capabilities.

#### Classification of incomplete genomes

Computing the probe frequencies uses only the coding regions of a genome sequence. It is now possible to obtain these coding sequences directly by using next generation sequencing technologies (NGS). Instead of completed whole genome sequences, the contigs of genomes or transcriptomes assembled by NGS can be the source data for the probe-set method. There are more than two thousand whole genome sequencing (WGS) projects, and over half of the genomes handled by them are still in the contig form (http://www.ncbi.nlm.nih.gov/genbank/wgs.html). This situation is probably caused by the fact that sequence assembly is a highly complex problem and may be the current bottleneck of WGS. Without well assembled genome sequences, conventional whole genome comparison methods, such as whole genome alignments, might not be applicable. We have proven that the probe-set method can highly correctly classify incomplete genome sequences (Figure 8). Besides, this method possesses an order-independent nature, which means that even the order of contigs of a genome is unknown, the probe-set frequencies of the genome can still be accurately obtained. Thus, as the NGS and WGS fields continue to increase the number of microorganisms being sequenced, the proposed method can be a useful method for the phylogenetic or functional analyses of organisms either with or *without *complete genome sequences.

#### Tree comparison method

The tree comparison method designed in this study measures the topological similarity between classification trees. We suppose that this method can be utilized as a standard procedure for tree comparisons. To measure the similarity/difference between phylogenetic trees constructed with different methods, or to quantify how well a tree reconstructs the relationships in phylogeny, metabolism or community among organisms involves tree comparisons. Previously, such comparisons were often done manually, lacking a quantitative measure. However, manual comparison is applicable only when the number of organisms is small. The tree comparison method that we developed is well applicable for big trees with hundreds, thousands or more organisms, facilitating the development and evaluation of future tree construction methods. For instance, in the HGT-removing experiment (Figure 6), where trees constructed with and without HGT genes were subjected to our tree comparison procedure, the similarities between trees were clearly revealed.

### Future work

At present we utilized all available probes provided by the Prosite, except for the highly frequent ones (as annotated by Prosite) to develop our probe-set based classification method. However, it is likely that some probes contribute little to the classification power of the proposed method. We have planned to establish a reduced version of the current probe-set by perhaps removing some highly infrequent probes or performing some critical factor analyses to identify the probes that exert major effects on the classification power. In addition to classifying microorganisms, the probe-set may also be applied to the identification of microorganisms with specific metabolic properties or phenotypes. For example, two microbial groups with opposite biological characteristics, e.g., nitrogen-fixing and non-nitrogen-fixing organisms, can be put together to compare their probe frequencies. The probes with significant difference in occurring frequency between the two groups may serve as good markers for detecting the nitrogen-fixing ability of other organisms.

So far, we have not considered the non-coding region of a genome when implementing the probe-set method. However, many regulatory sequences in a genome are not translated into proteins while they may still be functionally and evolutionarily important. For instance, some RNAs, such as ribozymes, act as enzymes. The 5' and 3' untranslated regions of messenger RNAs often contain sequence conserved regulatory elements [52]. Integrating these regulatory sequences and functional non-coding RNAs to the probe-set expands the source of information for species classification from the proteome level to the transcriptome level. This expansion is supposed to improve the comprehensiveness of the classifications.

## Conclusions

A classification method has been purposed to classify microbial genomes. A set of probes (i.e., conserved amino acid sequences with known biological functions) are used to encode microbial genomes into frequency patterns. The classification is achieved by hierarchical clustering of these frequency patterns. The method itself is a kind of compositional analysis which features computational inexpensiveness and high fault tolerance. This method can classify hundreds of genomes in minutes. Its classification results agree well with the phylogenetic relationships of microorganisms at higher classification levels, and clearly reflect the functional similarities among microorganisms at finer classification levels. Importantly, complete genome sequences are not the requisite for our method to obtain reliable results. In this post genomic era, when the amount of genome sequence data increases so rapidly, the high efficiency and novelty of the proposed method make it feasible for large scale classifications of microorganisms and phylogenetic studies of species with similar metabolic properties or incomplete genome sequences.

## Methods

### Data preparation

All proteomes were downloaded from the NCBI RefSeq server (http://www.ncbi.nlm.nih.gov/refseq/). Taxonomic data were retrieved from the NCBI Taxonomy database (http://www.ncbi.nlm.nih.gov/Taxonomy/). Amino acid sequence pattern descriptors provided by the Prosite database (http://au.expasy.org/prosite/) were utilized as the probes. Highly frequent sequence patterns, as annotated by the Prosite, were eliminated. The enzymatic categories, protein sequences and metabolic pathway information of enzymes were downloaded from the KEGG database (http://www.genome.jp/kegg/). The metabolic, biochemical and environmental characteristics of organisms were obtained from the GOLD database (http://www.genomesonline.org/). Horizontally transferring genes were downloaded from the HGT database (http://genomes.urv.cat/HGT-DB/). There are 415 microorganisms recorded in the HGT database; all these organisms were used in our HGT removal experiment.

### Random truncation of proteomes

For each "class" containing three or more organisms, three organisms were randomly selected to perform the random truncation experiment (Figure 8). For each selected organism, the contig sequences of its genome were randomly removed to a given truncation rate. At every truncation rate, the random removal was performed for 10 times, after each of which a classification tree of the selected organisms was reconstructed and compared with the tree that was constructed without the random removal of coding sequences. The correlation coefficient values (see the last subsection) shown in Figure 8 were the average value of the 10 repeated experiments.

### Computation of probe-set frequencies and the similarities of probe-set frequency patterns

The frequency of a probe for the proteome of a given species was determined by calculating the occurrence of the probe, which was normalized by dividing it by the number of protein-coding genes in the proteome. A probe-set frequency pattern is the collection of the normalized frequencies of all utilized probes. The similarity between two probe-set patterns was measured as the correlation coefficient (CC) between them by using the formula shown below,

(1) $$CC=\frac{\sum_{i=1}^{n}\left(f_{i}^{A}-\bar{f^{A}}\right)\left(f_{i}^{B}-\bar{f^{B}}\right)}{\sqrt{\sum_{i=1}^{n}\left(f_{i}^{A}-\bar{f^{A}}\right)}\sqrt{\sum_{i=1}^{n}\left(f_{i}^{B}-\bar{f^{B}}\right)}}$$

where $f_{i}^{A}$ and $f_{i}^{B}$ represent the frequencies of probe *i *in organism *A *and organism *B*, respectively, and *n *denotes the number of probes.

### Visualization of probe-sets

To visualize the probe-set frequency pattern of an organism, as shown in Figures 1, 2, 3, the frequency of each probe was represented by a small color-filled cell. The color-coded probe frequencies of the organism were then lined up in a row. For visual comparisons, the color-coded rows of multiple organisms were placed side by side, forming a color-coded band, in which the colors of cells in every column were standardized according to the following formula,

(2) $$f_{i}^{\prime }=\frac{f_{i}-\mu }{\sigma }$$

where *f_i _*and *f_i_' *respectively represent the raw and standardized frequency values of organism *i*, and *μ *and *σ *are the mean and standard deviation of the frequency values for all organisms in the column. A cell with a zero *f_i_' *is colored black. Positive and negative *f_i_' *values are represented by red and green colors, respectively, and the brightness of the color is in proportion to the absolute value of *f_i_'*.

When performing classification, the color-coded rows were clustered in a way described in the next subsection. After the clustering, similar color-coded rows should be placed close to one another.

### Construction of classification trees

In this study, the probe frequency patterns of organisms were clustered by the CLUSTER 3.0 program (http://bonsai.hgc.jp/~mdehoon/software/cluster/software.htm) to obtain the classification tree. The Spearman rank correlation, the nonparametric versions of the Pearson correlation coefficient, was used as the distance measure of probe-set patterns. This measurement worked more robust than Pearson correlation coefficient on reducing the effects of outliers. When the distance matrix was calculated, CLUSTER 3.0 worked based on the average linkage clustering algorithm in which the average of pairwise distances in two clusters is used to build the hierarchical clustering tree.

### Comparison of classification trees

Each organism in a classification tree can be considered a leaf of the tree. A traveling distance method is purposed here to describe the distance between leaves in the same rooted tree. For any two leaves, there exists a lowest common ancestor [53], as shown in Figure 7a. Given two leaves X and Y, starting from X and Y respectively, if there are *m *and *n *nodes to be traversed before reaching their lowest common ancestor, then the traveling distance between X and Y is computed as *m *+ *n*. For a tree with *N *organisms, an *N *× *N *leaf-to-leaf distance matrix can thus be obtained (see Figure 7b). To measure the similarity between trees, the CC of two matrices is calculated. A high CC value stands for a high similarity in topology for the compared trees. Because the computation of a CC value requires paired data, a limitation of this method is that the two trees under comparison should possess exactly the same nodes.

## Competing interests

The authors declare that they have no competing interests.

## Authors' contributions

CCL and WCL designed and carried out this study and drafted the manuscript. SML helped analyze classification data. PCL, CYT and YPPC conceived of the study, participated in its design and helped draft the manuscript. All authors read and approved the final manuscript.

## Supplementary Material

Additional file 1 **Large-scale proteomic tree**. There are 843 microbes included in this large-scale proteomic tree. The blue dotted line indicates that *Archaea *(lower part) and *Bacteria *(upper part) are separated by the probe-set clustering. Organisms of different phyla are labeled with different colors. The color codes are shown at the upper right corner of this figure. Click here for file

Additional file 2 **The HGT^+/- ^proteomic trees**. This experiment involves the 415 microorganisms recorded in the HGT database that are known to possess horizontally transferred genes [44]. (a) Tree HGT^+^: the proteomic tree constructed using whole genomes. (b) HGT^-^: the proteomic tree constructed with horizontally transferred genes removed. For clarity, Tree HGT^+ ^is divided into several large clusters, each of which is painted with a unique color. The color of a species in Tree HGT^- ^is given according to the color of the species in Tree HGT^+^. Clearly, the color patterns of both trees are very similar. Indeed, the correlation coefficient between these two trees is very high (0.93), indicating a high similarity in topology between these trees. Click here for file

## References

[B1] Cardenas E Tiedje JM New tools for discovering and characterizing microbial diversity Curr Opin Biotechnol 2008 19 6 544 549 10.1016/j.copbio.2008.10.010 18984052

[B2] Brodie EL DeSantis TZ Joyner DC Baek SM Larsen JT Andersen GL Hazen TC Richardson PM Herman DJ Tokunaga TK Application of a high-density oligonucleotide microarray approach to study bacterial population dynamics during uranium reduction and reoxidation Appl Environ Microbiol 2006 72 9 6288 6298 10.1128/AEM.00246-06 16957256 PMC1563607

[B3] Hugenholtz P Tyson GW Blackall LL Design and evaluation of 16S rRNA-targeted oligonucleotide probes for fluorescence in situ hybridization Methods Mol Biol 2002 179 29 42 11692872 10.1385/1-59259-238-4:029

[B4] Pace NR Stahl DA Lane DJ Olsen GJ The analysis of natural microbial-populations by Ribosomal-Rna sequences Adv Microb Ecol 1986 9 1 55

[B5] Sogin ML Morrison HG Huber JA Mark Welch D Huse SM Neal PR Arrieta JM Herndl GJ Microbial diversity in the deep sea and the underexplored "rare biosphere" Proc Natl Acad Sci USA 2006 103 32 12115 12120 10.1073/pnas.0605127103 16880384 PMC1524930

[B6] Symonds J Bergey Manual of Systematic Bacteriology, Krieg, Nr, Holt, Jg Lancet 1984 2 8411 1075 1076

[B7] Woese CR Bacterial evolution Microbiol Rev 1987 51 2 221 271 2439888 10.1128/mr.51.2.221-271.1987 PMC373105

[B8] Woese CR Fox GE Phylogenetic structure of prokaryotic domain - primary kingdoms Proc Natl Acad Sci USA 1977 74 11 5088 5090 10.1073/pnas.74.11.5088 270744 PMC432104

[B9] Woese CR Kandler O Wheelis ML Towards a natural system of organisms - proposal for the domains archaea, bacteria, and eucarya Proc Natl Acad Sci USA 1990 87 12 4576 4579 10.1073/pnas.87.12.4576 2112744 PMC54159

[B10] Lin J Gerstein M Whole-genome trees based on the occurrence of folds and orthologs: implications for comparing genomes on different levels Genome Res 2000 10 6 808 818 10.1101/gr.10.6.808 10854412 PMC310900

[B11] Qi J Wang B Hao BI Whole proteome prokaryote phylogeny without sequence alignment: A K-string composition approach J Mol Evol 2004 58 1 1 11 10.1007/s00239-003-2493-7 14743310

[B12] Mazurie A Bonchev D Schwikowski B Buck GA Phylogenetic distances are encoded in networks of interacting pathways Bioinformatics 2008 24 22 2579 2585 10.1093/bioinformatics/btn503 18820265 PMC2579716

[B13] Chang CW Lyu PC Arita M Reconstructing phylogeny from metabolic substrate-product relationships BMC Bioinformatics 2011 12 Suppl 1 S27 10.1186/1471-2105-12-S1-S27 21342557 PMC3044282

[B14] Borenstein E Kupiec M Feldman MW Ruppin E Large-scale reconstruction and phylogenetic analysis of metabolic environments Proc Natl Acad Sci USA 2008 105 38 14482 14487 10.1073/pnas.0806162105 18787117 PMC2567166

[B15] Clemente JC Satou K Valiente G Reconstruction of phylogenetic relationships from metabolic pathways based on the enzyme hierarchy and the gene ontology Genome Inform 2005 16 2 45 55 16901088

[B16] Clemente JC Satou K Valiente G Phylogenetic reconstruction from non-genomic data Bioinformatics 2007 23 2 E110 E115 10.1093/bioinformatics/btl307 17237077

[B17] Forst CV Schulten K Phylogenetic analysis of metabolic pathways J Mol Evol 2001 52 6 471 489 11443351 10.1007/s002390010178

[B18] Shigenobu S Watanabe H Hattori M Sakaki Y Ishikawa H Genome sequence of the endocellular bacterial symbiont of aphids Buchnera sp APS Nature 2000 407 6800 81 86 10.1038/35024074 10993077

[B19] Stephens RS Kalman S Lammel C Fan J Marathe R Aravind L Mitchell W Olinger L Tatusov RL Zhao QX Genome sequence of an obligate intracellular pathogen of humans: Chlamydia trachomatis Science 1998 282 5389 754 759 9784136 10.1126/science.282.5389.754

[B20] Durot M Bourguignon PY Schachter V Genome-scale models of bacterial metabolism: reconstruction and applications FEMS Microbiol Rev 2009 33 1 164 190 10.1111/j.1574-6976.2008.00146.x 19067749 PMC2704943

[B21] Cash P Proteomics in the study of the molecular taxonomy and epidemiology of bacterial pathogens Electrophoresis 2009 30 Suppl 1 S133 S141 19517493 10.1002/elps.200900059

[B22] Jabbour RE Deshpande SV Stanford MF Wick CH Zulich AW Snyder AP A protein processing filter method for bacterial identification by mass spectrometry-based proteomics J Proteome Res 2011 10 2 907 912 10.1021/pr101086a 21126090

[B23] Trost B Haakensen M Pittet V Ziola B Kusalik A Analysis and comparison of the pan-genomic properties of sixteen well-characterized bacterial genera BMC Microbiol 2010 10 258 10.1186/1471-2180-10-258 20942950 PMC3020658

[B24] Turse JE Marshall MJ Fredrickson JK Lipton MS Callister SJ An empirical strategy for characterizing bacterial proteomes across species in the absence of genomic sequences PLoS One 2010 5 11 e13968 10.1371/journal.pone.0013968 21103051 PMC2980473

[B25] Ciccarelli FD Toward automatic reconstruction of a highly resolved tree of life Science 2006 311 5765 1283 1287 10.1126/science.1123061 16513982

[B26] Marchler-Bauer A Lu SN Anderson JB Chitsaz F Derbyshire MK DeWeese-Scott C Fong JH Geer LY Geer RC Gonzales NR CDD: a Conserved Domain Database for the functional annotation of proteins Nucleic Acids Res 2011 39 D225 D229 10.1093/nar/gkq1189 21109532 PMC3013737

[B27] Bernal A Ear U Kyrpides N Genomes OnLine Database (GOLD): a monitor of genome projects world-wide Nucleic Acids Res 2001 29 1 126 127 10.1093/nar/29.1.126 11125068 PMC29859

[B28] Pruitt KD Tatusova T Maglott DR NCBI reference sequences (RefSeq): a curated non-redundant sequence database of genomes, transcripts and proteins Nucleic Acids Res 2007 35 Database D61 D65 17130148 10.1093/nar/gkl842 PMC1716718

[B29] Boore JL Brown WM Big trees from little genomes: mitochondrial gene order as a phylogenetic tool Curr Opin Genet Dev 1998 8 6 668 674 10.1016/S0959-437X(98)80035-X 9914213

[B30] Brown JR Douady CJ Italia MJ Marshall WE Stanhope MJ Universal trees based on large combined protein sequence data sets Nat Genet 2001 28 3 281 285 10.1038/90129 11431701

[B31] Li W Fang W Ling L Wang J Xuan Z Chen R Phylogeny based on whole genome as inferred from complete information set analysis J Biol Phys 2002 28 3 439 447 10.1023/A:1020316706928 PMC3456743 23345787

[B32] Snel B Bork P Huynen MA Genome phylogeny based on gene content Nat Genet 1999 21 1 108 110 10.1038/5052 9916801

[B33] McHardy AC Rigoutsos I What's in the mix: phylogenetic classification of metagenome sequence samples Curr Opin Microbiol 2007 10 5 499 503 10.1016/j.mib.2007.08.004 17933580

[B34] Hansen AK Moran NA Aphid genome expression reveals host-symbiont cooperation in the production of amino acids Proc Natl Acad Sci USA 2011 108 7 2849 2854 10.1073/pnas.1013465108 21282658 PMC3041126

[B35] Wilson ACC Ashton PD Calevro F Charles H Colella S Febvay G Jander G Kushlan PF Macdonald SJ Schwartz JF Genomic insight into the amino acid relations of the pea aphid, Acyrthosiphon pisum, with its symbiotic bacterium Buchnera aphidicola Insect Mol Biol 2010 19 249 258 20482655 10.1111/j.1365-2583.2009.00942.x

[B36] Zientz E Dandekar T Gross R Metabolic interdependence of obligate intracellular bacteria and their insect hosts Microbiology and Molecular Biology Reviews 2004 68 4 745 770 10.1128/MMBR.68.4.745-770.2004 15590782 PMC539007

[B37] Kisand V Wikner J Limited resolution of 16S rDNA DGGE caused by melting properties and closely related DNA sequences J Microbiol Methods 2003 54 2 183 191 10.1016/S0167-7012(03)00038-1 12782374

[B38] Vandamme P Pot B Gillis M DeVos P Kersters K Swings J Polyphasic taxonomy, a consensus approach to bacterial systematics Microbiological Reviews 1996 60 2 407 438 8801440 10.1128/mr.60.2.407-438.1996 PMC239450

[B39] Kyrpides NC Genomes OnLine Database (GOLD 1.0): a monitor of complete and ongoing genome projects world-wide Bioinformatics 1999 15 9 773 774 10.1093/bioinformatics/15.9.773 10498782

[B40] Stiles ME Holzapfel WH Lactic acid bacteria of foods and their current taxonomy Int J Food Microbiol 1997 36 1 1 29 10.1016/S0168-1605(96)01233-0 9168311

[B41] Boussau B Gueguen L Gouy M Accounting for horizontal gene transfers explains conflicting hypotheses regarding the position of aquificales in the phylogeny of Bacteria BMC Evolutionary Biology 2008 8 272 10.1186/1471-2148-8-272 18834516 PMC2584045

[B42] Dutta C Pan A Horizontal gene transfer and bacterial diversity J Biosci 2002 27 1 27 33 10.1007/BF02703681 11927775

[B43] Kanhere A Vingron M Horizontal Gene Transfers in prokaryotes show differential preferences for metabolic and translational genes BMC Evolutionary Biology 2009 9 9 10.1186/1471-2148-9-9 19134215 PMC2651853

[B44] Garcia-Vallve S Guzman E Montero MA Romeu A HGT-DB: a database of putative horizontally transferred genes in prokaryotic complete genomes Nucleic Acids Res 2003 31 1 187 189 10.1093/nar/gkg004 12519978 PMC165451

[B45] Coram NJ Rawlings DE Molecular relationship between two groups of the genus Leptospirillum and the finding that Leptosphillum ferriphilum sp nov dominates South African commercial biooxidation tanks that operate at 40 degrees C Appl Environ Microbiol 2002 68 2 838 845 10.1128/AEM.68.2.838-845.2002 11823226 PMC126727

[B46] Tyson GW Lo I Baker BJ Allen EE Hugenholtz P Banfield JF Genome-directed isolation of the key nitrogen fixer Leptospirillum ferrodiazotrophum sp nov from an acidophilic microbial community Appl Environ Microbiol 2005 71 10 6319 6324 10.1128/AEM.71.10.6319-6324.2005 16204553 PMC1266007

[B47] Sekiguchi Y Muramatsu M Imachi H Narihiro T Ohashi A Harada H Hanada S Kamagata Y Thermodesulfovibrio aggregans sp nov and Thermodesulfovibrio thiophilus sp nov., anaerobic, thermophilic, sulfate-reducing bacteria isolated from thermophilic methanogenic sludge, and emended description of the genus Thermodesulfovibrio Int J Syst Evol Micr 2008 58 2541 2548 10.1099/ijs.0.2008/000893-0 18984690

[B48] Dopson M Baker-Austin C Bond PL First use of two-dimensional polyacrylamide gel electrophoresis to determine phylogenetic relationships J Microbiol Meth 2004 58 3 297 302 10.1016/j.mimet.2004.04.007 15279933

[B49] Delcher AL Kasif S Fleischmann RD Peterson J White O Salzberg SL Alignment of whole genomes Nucleic Acids Res 1999 27 11 2369 2376 10.1093/nar/27.11.2369 10325427 PMC148804

[B50] Lu CL Huang YL Huang CC Tang CY SoRT(2): a tool for sorting genomes and reconstructing phylogenetic trees by reversals, generalized transpositions and translocations Nucleic Acids Res 2010 38 W221 W227 10.1093/nar/gkq520 20538651 PMC2896082

[B51] Colwell RR Polyphasic Taxonomy of Genus Vibrio - Numerical Taxonomy of Vibrio-Cholerae, Vibrio-Parahaemolyticus, and Related Vibrio Species Journal of Bacteriology 1970 104 1 410 433 5473901 10.1128/jb.104.1.410-433.1970 PMC248227

[B52] Repoila F Darfeuille F Small regulatory non-coding RNAs in bacteria: physiology and mechanistic aspects Biol Cell 2009 101 2 117 131 10.1042/BC20070137 19076068

[B53] Aho AV Hopcroft JE Ullman JD On finding lowest common ancestors in trees Proceedings of the fifth annual ACM symposium on Theory of computing 1973 Austin: ACM 253 265

