# Supplementary figures and images for: Metabolic classification of microbial genomes using functional probes
Source: BMC Genomics. 2012 Apr 27;13:157. doi: 10.1186/1471-2164-13-157 (PMC3355368; doi:10.1186/1471-2164-13-157)

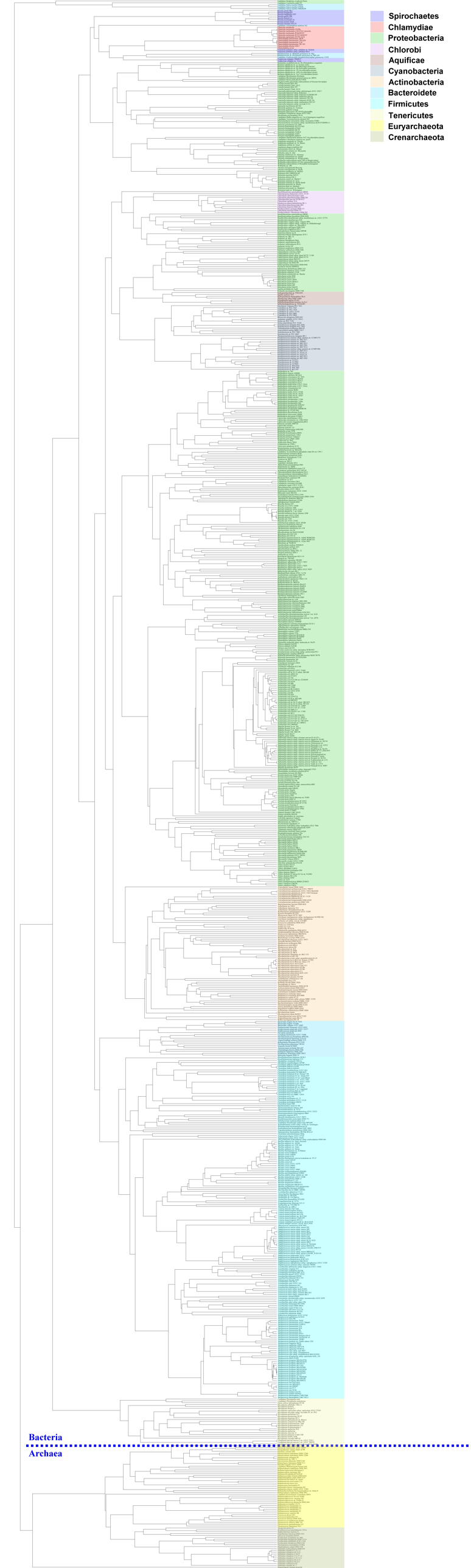

Supplement: Additional file 1 — Large-scale proteomic tree. There are 843 microbes included in this large-scale proteomic tree. The blue dotted line indicates that Archaea (lower part) and Bacteria (upper part) are separated by the probe-set clustering. Organisms of different phyla are labeled with different colors. The color codes are shown at the upper right corner of this figure. [file 1471-2164-13-157-S1.JPEG]

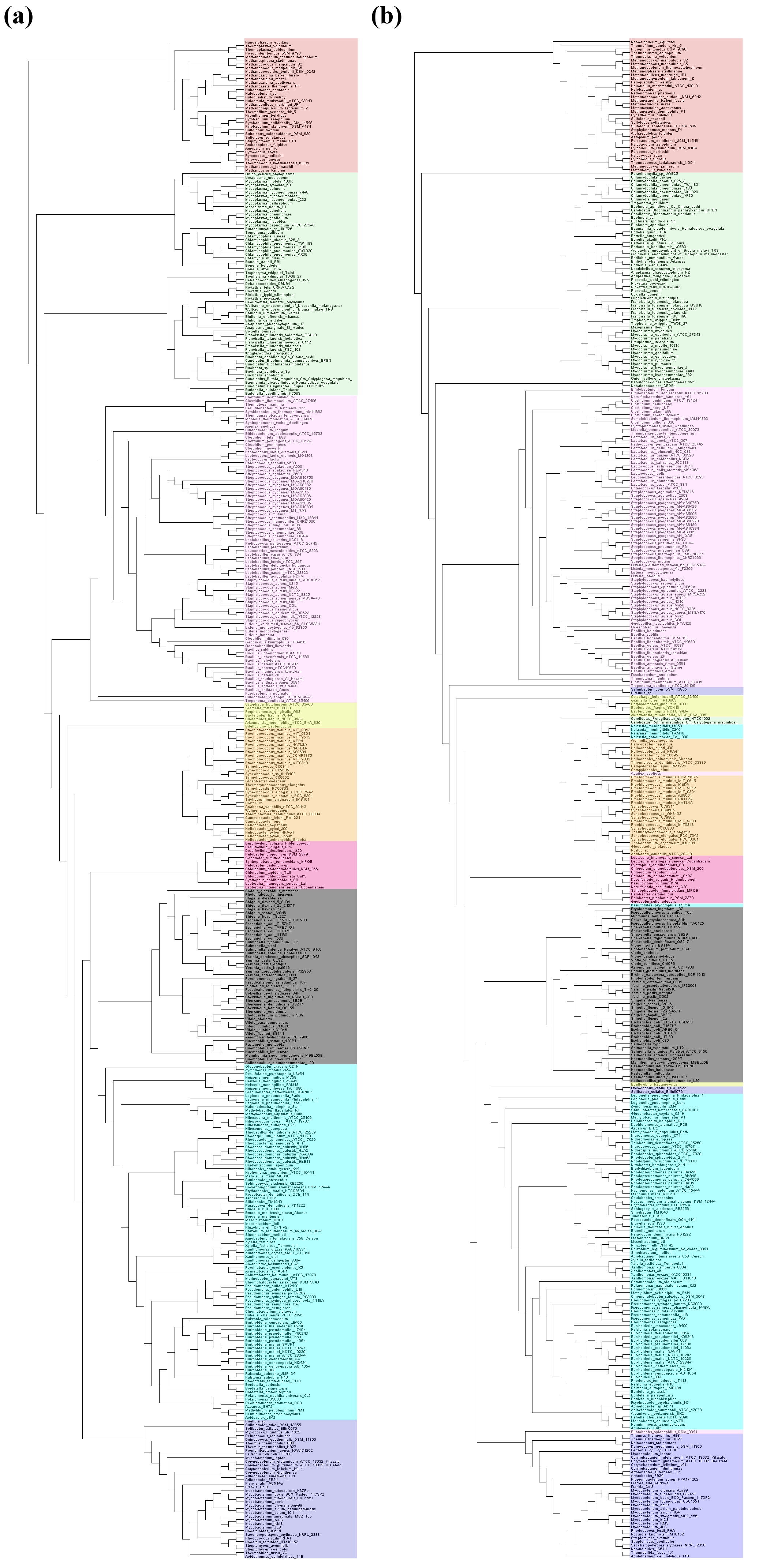

Supplement: Additional file 2 — The HGT+/- proteomic trees. This experiment involves the 415 microorganisms recorded in the HGT database that are known to possess horizontally transferred genes [44]. (a) Tree HGT+: the proteomic tree constructed using whole genomes. (b) HGT-: the proteomic tree constructed with horizontally transferred genes removed. For clarity, Tree HGT+ is divided into several large clusters, each of which is painted with a unique color. The color of a species in Tree HGT- is given according to the color of the species in Tree HGT+. Clearly, the color patterns of both trees are very similar. Indeed, the correlation coefficient between these two trees is very high (0.93), indicating a high similarity in topology between these trees. [file 1471-2164-13-157-S2.JPEG]
